# Investigation of the conditions for continuous information conveyance by two autonomous conversational agents

**DOI:** 10.3389/frobt.2025.1417488

**Published:** 2025-04-17

**Authors:** Takamichi Isowa, Kohei Ogawa, Satoshi Sato, Tomonori Kubota, Hiroshi Ishiguro

**Affiliations:** ^1^ Department of Systems Innovation, Graduate School of Engineering Science, Osaka University, Osaka, Japan; ^2^ Department of Information and Communication Engineering, Graduate School of Engineering, Nagoya University, Nagoya, Japan

**Keywords:** system of conveying information, knowledge representation, multiple conversational agents, passive-social conversation, conversation generation system

## Abstract

**Introduction:**

In recent years, information conveyance through conversation using agents such as robots and avatars has gained attention. Among them, conversation by two agents has been shown to encourage effective information conveyance. Previous studies have also demonstrated that incorporating subjective information, such as emotions, into conversations enhances this effect. Therefore, a medium for information conveyance involving two autonomous agents and including subjective information is expected to be effective.

**Methods:**

In this study, such a medium was implemented, and the conditions necessary for it to convey information continuously were investigated. Objective information was defined as the content of existing news, and subjective information was defined as the preference toward the news. A frame structure was used for organizing objective information, and a network structure was used for subjective information. A method was developed to autonomously obtain both types of information. This knowledge was then distributed to two agents, who exchanged it and attempted to understand each other through conversation.

**Results:**

Experiments were conducted to determine whether the subjective information obtained autonomously by the agents was as natural and consistent as that of humans. Further experiments examined the conditions for enabling continuous information conveyance using the medium.

**Discussion:**

The results indicated that conveying important information first and using robots rather than text were effective strategies for maintaining continuous information conveyance.

## 1 Introduction

Until now, a variety of information has been conveyed through various media such as videos, images, and texts. With the development of technology, information conveyance through robots, avatars, and chatbots has become possible. These agents are gaining attention in recent years because they can convey information through conversation and non-verbal methods. Pepper by SoftBank and Alexa by Amazon are prime examples of agents that convey information. Thus, systems where agents convey information through conversation or non-verbal methods are widely accepted in society.

Among the methods of information conveyance by agents, a passive-social conversation has been proposed, in which information is conveyed by conversations between two agents (Hayashi et al., 2007). When applied to robots, this method has been shown to attract the attention and convey information effectively (Hayashi et al., 2008). In addition, it has been shown that conversations between two agents and a person can increase the persuasive effect (Kantharaju et al., 2018) and attract interest in the conversation content more easily (Kantharaju et al., 2018). Therefore, it is considered that information conveyance through conversations using two agents can be highly effective.

In human-to-human conversations, it is known that verbalizing and expressing one’s emotions is important for interpersonal relationships (Clark and Reis, 1988a; Fussell, 2002) and in dialogue systems, the effectiveness of emotional intervention during conversations has been reported in a previous study (Partala and Surakka, 2004a). In situations requiring persuasion and negotiation, it has also been shown that expressing one’s emotions during conversations is effective (Keltner and Haidt, 1999; Morris and Keltner, 2000). Therefore, in information conveyance through conversations, conveying information with one’s own emotions is considered to convey information effectively.

Based on the above-mentioned points, information conveyance using two agents with subjective information, such as emotions, can be effective. A similar system has been presented by Iio et al. (2017), in which autonomous multi-agents interact with humans while expressing emotions. The effectiveness of these systems, which include passive-social conversations by robots, has been verified but these studies have not investigated on which type of conversation is preferred. Therefore, when realizing two autonomous agents that convey information in a conversational format, including subjective information, it is necessary to construct a system that can easily control the conversation flow and offer various outputs for investigating the preferred type of conversation. The aim of this study is to realize such a medium with two agents and investigate what type of conversations are preferable that can convey information continuously.

To realize a medium using two autonomous agents that convey information in a conversational format, including subjective information, a technology that can convert information with subjective elements into a conversation is required. With regards to conversation generation technology, the emergence of large language models such as the GPT series has made it possible to generate natural sentences. However, the output can change significantly depending on the prompt, and as mentioned above, controlling the flow of the conversation can become difficult. Therefore, in this research, the conversion of information into conversation has been achieved using a rule-based method, where information is inputted into prepared conversation templates.Furthermore, the expression of subjective information by the two agents is achieved by introducing word preference levels. The overall preference level of the conveyed information is calculated based on the preference levels of the words included in the information.

Humans generally have consistent emotions and attitudes that tend to persist (Rocklage and Luttrell, 2021). Therefore, if agents represent inconsistency in subjective information, such as emotions, or if their past opinions contradict their current ones, people are likely to feel uncomfortable. This inconsistency could lead people to refuse receiving information. Therefore, to realize an information conveyance medium that expresses information, including subjective information, as a conversation, it is required for the subjective information given by the agents to be consistent. This has been investigated through experiments in this study.

The information conveyance medium using two agents in this study must not only be realized but also be capable of continuously conveying information without losing the recipient’s interest.

For effective information conveyance, the medium must first attract the recipient’s attention. Once attention is attracted, it is crucial that the recipient remains engaged with the medium and continues receiving information. Previous research has shown that the presence of two robots naturally attracts attention. Therefore, this study focuses particularly on continuous information conveyance.

Accordingly, this study aims to implement an information conveyance medium using two agents and identify the conditions necessary for recipients to continuously receive information. Specifically, we consider two key factors that enable continuous information conveyance: 1) the ease of understanding the information provided by the medium and 2) the medium’s ability to sustain the recipient’s interest actively. We define these as the clarity of the information provided by the medium and the sustained attractiveness of the medium, respectively. To enhance these indicators, we conducted experiments to examine the conditions that contribute to their improvement.

This research aims to realize an information conveyance medium with subjective information by two autonomous agents. The purpose of this study is to investigate the conditions necessary for this medium to continuously convey information. To realize the medium, an experiment has been performed to verify whether the agents can obtain consistent subjective information. Furthermore, to verify the hypotheses about the conditions necessary for the medium to convey information continuously, two subject experiments have been performed.

In this paper, Section 2 gives an overview of the present study in the context of the existing research, whereas Section 3 explains the implementation method for a medium that converts information with subjective elements into a conversational format. Section 4 describes the aforementioned experiments in detail and the results obtained from them. The conclusion drawn from this study, the limitations, and points for future work have been discussed in Section 5.

## 2 Related work

To realize an information conveyance medium that includes subjective information through conversations between two agents, in this section, we discuss previous research related to the technology of conversational agents and information conveyance medium.

### 2.1 Conversational agent

The technology of conversational agents can be divided into two categories: 1) multiple conversational agents, where multiple agents communicate with each other or with humans and 2) conversational agent where a single agent engages in conversations with a human. For each category, we discuss the techniques and insights required for the two autonomous conversational agents that have been proposed in this study. Furthermore, we explain the effects of expressing subjective information, such as emotions, during conversations with agents.

#### 2.1.1 Multiple conversational agents

When advancing research on multiple conversational agents, it is crucial to build upon existing knowledge of human-to-human interaction. Numerous studies have been conducted on human conversation from the perspectives of sociolinguistics and neurolinguistics. From a sociolinguistic perspective, it has been shown that dialogue involving multiple speakers enhances the accuracy of information conveyance compared to monologue-style communication (Fox Tree, 1999). From a neurolinguistic perspective, studies have revealed distinct brain activity patterns in triadic interactions and collaborative situations compared to dyadic interactions. Specifically, as the number of participants increases, higher-order neural networks responsible for inferring others’ intentions and thoughts become more active, leading to observed synchronization in brain activity (Xie et al., 2020; Nozawa et al., 2016).

Research applying these human conversation insights to agents and robots has shown that two robots are more effective in attracting human attention than a single robot (Hayashi et al., 2007). Additionally, robotic comedy performances have been found to achieve a level of naturalness comparable to human performances (Hayashi et al., 2008), with an overall impression that favored the robotic performances. Furthermore, studies indicate that when humans make conversations with agents or robots, the presence of multiple agents enhances persuasive effects, increases the interest in conversation topics, and facilitates more sustained communication (Kantharaju et al., 2018; Nishimura et al., 2013; Arimoto et al., 2018).

These researches suggest that dialogue involving multiple agents or robots, as opposed to a single-agent interaction, encourages participants to actively infer others’ intentions and thoughts, thereby promoting richer information exchange and enabling more sustained and effective information conveyance. However, previous studies using passive social dialogue with robots and agents relied on pre-scripted content. In this study, we aim to develop two agents capable of autonomously acquiring information and making conversation about that information.

#### 2.1.2 Single conversational agent

Various studies have been performed on single conversational agent, including research on the chatbot dialogue system and human-robot dialogue system. The technology of a single conversational agent can be categorized into task-oriented dialogue system, which focuses on executing a predetermined task, and non-task-oriented dialogue system, which does not aim for a task but instead engages in casual conversation with the user.

The task-oriented dialogue systems were once often implemented using rule-based approaches (Green et al., 1961; Winograd, 1971). Since the emergence of neural networks, using RNNs and LSTMs, it has became possible to respond based on the context by extracting semantic information across multiple sentences. Recently, using pretrained language models based on the Transformer (Vaswani et al., 2017), responses that consider even longer contexts have been realized by fine-tuning for downstream tasks as required. In addition, task-oriented dialogue systems, such as Apple’s Siri and Amazon’s Alexa, have been applied in the real world, and many such services are already in use.

With regards to the non-task-oriented dialogue systems, many chatbots, such as Mitsuku (Worswick, 2018), Meena (Adiwardana et al., 2020), and Blenderbot (Roller et al., 2020), have been developed that have achieved human-like evaluation scores for short-turn dialogues. With the rise of large language models based on the Transformer, such as GPT-4 (OpenAI, 2023), PaLM2 (Anil et al., 2023), and LLaMA (Touvron et al., 2023), consistent dialogues in long contexts have become possible, and several services, including ChatGPT, are now being used on a global scale.

While large language models using Transformers can produce natural and human-like conversations, it is difficult for them to respond with specific emotions. Indeed, it has been reported that such multi-language models reflect Western culture in their outputs (Havaldar et al., 2023). This could lead the media to not work well when conveying information containing specific values or opinions. For the purpose of this research, which is to investigate the conditions for medium to convey information continuously, it is necessary to control the order of information in the generated conversations with ease. However, with models like GPT-4, the output can change significantly depending on the prompts (Gan and Mori, 2023), and thus control of the output conversations becomes difficult. These considerations suggest that rule-based methods, where templates of conversational texts are prepared and the information to be conveyed is fitted to these templates, are superior to large language models in that it is easier to control the output conversations and subjective information. To generate a conversation by connecting conversation templates, it is necessary to classify the templates according to patterns that are unlikely to cause conversational breakdowns, such as adjacency pairs (Schegloff and Sacks, 1973), and arrange them in a predetermined order. Therefore, by adopting a state transition model that transitions templates in an order unlikely to cause breakdowns, this method can be realized.

#### 2.1.3 Representation of subjective information in agent dialogue

Emotion is a key characteristic of human nature and plays a crucial role in shaping thoughts and behaviors in daily life. Emotions have a social nature, as they are elicited through interactions with others and expressed toward others. Consequently, numerous studies have been conducted on this aspect (Parkinson, 1996; Van Kleef, 2009). In particular, research on emotional expression in conversation has found that interactions on social media frequently exhibit emotional alignment and empathy between dialogue partners (Kim et al., 2012). Furthermore, expressions containing emotions have been shown to be more memorable than those without emotional content (Lee and Potter, 2020).

Since the proposal of affective computing (Picard, 2000), which involves machines handling emotions, research on the effectiveness of human-like conversations contained by emotions has been done in conversation systems. For example, there are reports in which conversational systems can reduce users’ stress through emotional interactions (Clark and Reis, 1988b) and the effectiveness of emotional interventions of conversational systems (Partala and Surakka, 2004b). Furthermore, recent proposals have introduced methods of representing emotional expressions in the output conversations using deep learning and reinforcement learning (Huang et al., 2018; Li et al., 2019).

Therefore, expressing emotions can also be effective in systems where two agents convey information through conversation. However, the researches mentioned above, the system responded to the user’s speech with emotional expressions and systems that proactively represent opinions or feelings about information or news have not been proposed to date. Therefore, this research aims to realize a system that constructs the agents’ unique subjective information that is to be conveyed and expresses it in a conversation.

Considering the multiple conversational agent, single conversational agent, and the representation of subjective information in the agent dialogue discussed above, a system where two agents autonomously obtain subjective information and generate conversations containing it using a rule-based approach can convey information effectively.

### 2.2 Media for information conveyance

It is known that consumers who receive information from the media have specific preferences and perceptions when choosing their media sources (Larkin, 1979). For instance, in the medical field, it is shown that conveying information to patients in an easily understandable way is crucial (Newton et al., 1998). Similarly, it can be considered important for the media to present information in a way that is easy to understand.

Furthermore, one of the important factors in media conveying information is the attractiveness of the medium. Social media, which is widely prevalent worldwide, is shown to attract users and provide an environment that retains them, thereby increasing the users’ engagement time in social media (Bedjaoui et al., 2018). Thus, it is important for media to attract interest continuously in order to acquire users.

Therefore, for the medium to be liked by users and be able to convey information continuously, the clarity of the information being provided by it and its ability to maintain the interest of users continuously are important. In this research, we investigate the conditions necessary for the realized medium to convey information continuously from the perspectives of the clarity of the information provided by the medium and the sustained attractiveness of the medium.

## 3 Systems for converting information into conversation

To realize a system where two autonomous agents convey information through conversations with subjective information, both agents must engage in the conversation and include objective as well as subjective information. Given that humans exchange a large amount of information through conversations (Dunbar et al., 1997), if the two agents could express information exchanges with conversations, the aforementioned system can be realized. For this, the two agents need to acquire information and then exchange it through conversations.

In this research, the system extracts information from existing news articles and uses it to construct a knowledge structure. That is further distributed to the two agents. These agents, referring to their own knowledge, complement the missing information by exchanging it through a conversation. The aim of this study is to convert the information into a conversation by following the above steps. Furthermore, with regards to the subjective information, the agents calculate how favorable the existing news articles are based on the knowledge they possess and acquire this in a numerical form. Similar to the objective information obtained from the news, as mentioned above, conversations about subjective information can be generated by representing the process of information exchange in a conversation.

The subsections below describe the implementation method for the aforementioned system via the following three points: 1) the form in which the information is held, 2) the procedure for acquiring the information required for the conversation, and 3) generating a conversation from the acquired information.

### 3.1 The form in which the information is held

As mentioned before, to realize the system, it is necessary to express the information as knowledge possessed by the two agents. For this, the information is divided into objective and subjective categories.

#### 3.1.1 Construction of objective information as knowledge

Objective information is converted to a frame structure. The frame structure is a method of knowledge representation consisting of attributes called slots and values called facets that belong to these slots. In this research, as shown on the left-hand side in Figure 2, the agents acquire knowledge by filling in facets that belong to the eight slots of “When,” “Where,” “Who,” “What,” “To what,” “Why,” “How,” and “Predicate.” For example, the “When” slot contains the facet “from October 1.” Slots such as the “How” in which no facet is filled in, behave as knowledge that the agent does not have, i.e., knowledge that the agent does not know of. By introducing such a frame structure, the number of slots remains consistent at eight. When generating a conversation, this makes it easy to control the order in which the different slots should be outputted.

#### 3.1.2 Construction of subjective information as knowledge

The two agents make conversations about a news article based on the objective information, as shown on the left-hand side in Figure 2. In this research, quantification is done of how favorably the agent feels about the news and this number is used as the subjective information. It is assumed that the preference toward the news depends on the words contained in it, and thus, the subjective information is constructed as follows. First, the agents maintain a preference for each commonly used word as a the preference scale, which is individually scored as a float value between −1.0 and 1.0. The current system has 18,600 words. Each of the two agents determines the overall preference of the news by calculating the average the preference scale of the words appearing in the news.

However, to achieve this, an algorithm that enables the agents to autonomously determine the preference scale for a large number of commonly used words is required. In this research, this issue has been solved by implementing a system where the preference scale for the remaining words is automatically calculated by tracing the conceptual network when the preference scale is set for some words out of many, using a network such as ConceptNet (Speer et al., 2017), precisely defines the conceptual structures between words.

**FIGURE 1:**
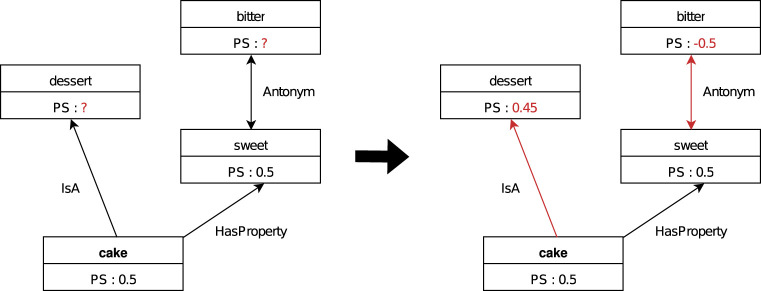
A simplified diagram focusing on the word “cake” in ConceptNet. The PS in the diagram represents the “Preference Scale”, a unique metric introduced in this study to indicate word preference information. Since ConceptNet defines relationships between words in a highly structured manner, propagating the “Preference Scale” based on these relationships enables the construction of a semantic network that aligns with human preferences.

ConceptNet has well-defined relationships between tens of thousands of words that are used daily. For example, if there are two words, “cake” and “dessert,” an inheritance relationship, called an “IsA” relationship, is defined, which expresses the fact that “a cake is a dessert.” In addition to this, there are 34 different types of relationships, such as the “HasA” relationship seen between “bird” and “feathers”, and the “PartOf” relationship seen between “Tokyo” and “Japan.” Here, for the words “cake” and “dessert” mentioned in the example, since the “IsA” relationship is defined between them, it is considered that if people have a favorable impression of “cake,” they would also tend to have a favorable impression of “dessert.” Therefore, if the preference scale of 0.5 is assigned to “cake,” it is desirable that a value close to 0.5 would also be propagated and assigned to “dessert” as shown in Figure 1.

Further, there is an “Antonym” relationship defined between the words “sweet” and “bitter,” signifying opposites. Unlike the previous example, if one has a favorable impression toward the word “sweet,” it can be expected that they will have an unfavorable impression toward the word “bitter.” Therefore, if the preference scale of 0.5 is assigned to “sweet,” it is desirable to propagate and assign a value close to −0.5 to “bitter” as shown in Figure 1.

Therefore, by setting the propagation rates according to the relationships, such as propagating the preference scale with a weight of 0.9 in the case of “IsA” relationships and a weight of −1.0 in the case of “Antonym” relationships, if the preference scale for a word has not been set, it can be automatically determined and propagated from the relationships with words already assigned the preference scale among those connected to it.

### 3.2 Obtaining knowledge

This section describes how to the objective and subjective information is obtained. The objective information is obtained by extracting the eight elements of information mentioned in Section 3.1.1 from news articles. In particular, the news article is first summarized into a single sentence. Then, a syntactic analysis of the extracted sentence is performed to determine which information belongs to which slot, and the facets are filled accordingly. In the following, we explain each step.

First, a summarization API is used to summarize the existing news articles into a single sentence. The reason for summarizing information into a single sentence is that if the summary spreads across multiple sentences, there is a possibility that more information than the eight elements, mentioned in Section 3.1.1, will be extracted. In this research, the COTOHA API, developed by NTT Communications Corporation (NTT, 2018), has been used. The COTOHA API is one of the most outstanding Japanese summarization APIs, capable of generative as well as extractive summarization.

Next, the system conducts a dependency analysis on the summarized sentence using KNP(Kawahara and Kurohashi, 2006). KNP can analyze and output which word is modifying which in Japanese, and if a word contains geographical or temporal information, it can output that as well. Thus, based on the location and time information outputted by KNP, the “When” and “Where” phrases are extracted. Further, the subject “Who,” the object “What,” and the complement “To what” are detected by KNP as phrases modifying the “Predicate” at the end of the sentence. Among the phrases modifying the “Predicate,” the system extracts “Why” and “How” from the form of the sentence.

For subjective information, as mentioned above, a human (annotator) sets the preference scale manually for a part of the many words used in daily life, and the preference scale propagates automatically to express the preferences for the other words as numerical values. The propagated value changes based on the initial manually inputted the preference scale, enabling different agents to express different values. Subsequently, the average of the preference scale for the group of words appearing in the eight elements of objective information is calculated. This is used in generating a conversation as the overall preference for the news.

### 3.3 Generating a conversation

**FIGURE 2:**
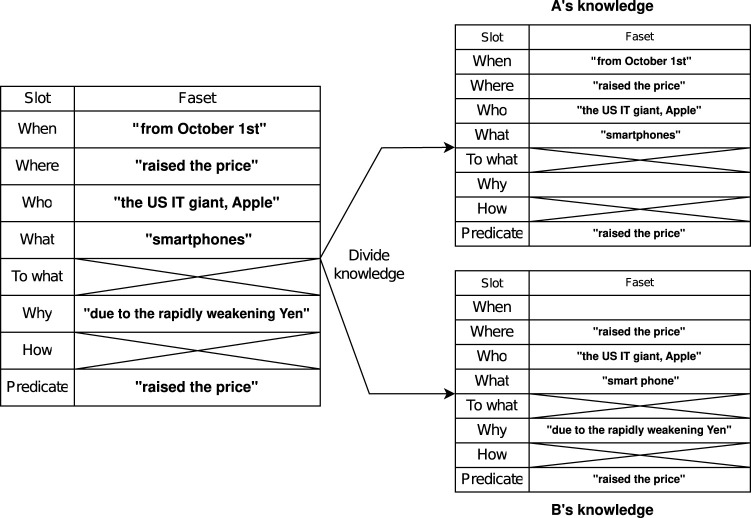
The information to be conveyed is represented as knowledge using a frame structure (as shown on the left-hand side). When making a conversation, knowledge is distributed to the two agents (as shown on the right-hand side diagram). Agents make conversations by asking the other for missing information or by presenting the information they possess.

By performing the above steps, the agents have obtained two types of information: structured knowledge with the structures consisting of eight components extracted from existing news articles, and the degree of preference toward the news. In the following, the procedure for generating a conversation using these two types of information is explained. In order to express the exchange of information that the two agents have as a conversation, each agent should hold independent knowledge, and thus the objective information is distributed to the two agents. As mentioned before, the subjective information does not require distribution because each agent holds a different value of the preference scale. As shown in Figure 2, the objective information is assigned so that either of the two agents hold the information. For example, if the acquired information is of the “When” type, either A or B, or both, need to hold this information of “When.” This is because if the information of “When” does not appear in the conversation even though it is in the original information, it cannot convey sufficient information.

**FIGURE 3:**
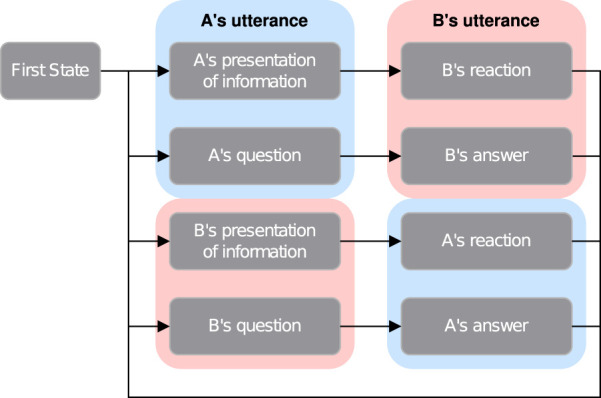
The state transition model of conversation. “A’s presentation of information” describes a state where agent A presents a certain facet from the frame. On the other hand, “B’s question” describes a state where agent B requests information to fill in the missing facets in the frame.

Next, using the state transition model shown in Figure 3, the agents determine the flow of the conversation. In Figure 3, each node represents the state of the conversation, in which the transition probabilities between states are individually set. The system that controls the conversation manages the state transitions and determines the current state acording to the transition probabilities. When the state is “presentation of information,” the agent presents one facet in the frame, and when the state is “question,” the agent requests information to fill in the missing facets in the frame.

Furthermore, template sentences such as “I heard it is ‘When”’ are defined for each of the eight elements of information and according to each conversation state. When the two agents present information, they generate conversation sentences by filling in the template sentences with facets. For instance, when the knowledge structure is distributed shown in the right-hand side of Figure 2 and Agent B presents the “When” information, the template sentence “I heard it is ‘When”’ is filled in and the output is similar to “I heard it is from October 1st.” For subjective information, the preference scale for the news is set with a float value between −1.0 and 1.0. Template sentences are set in increments of 0.25, and a conversation sentence is generated by outputting the corresponding template sentence.

**FIGURE 4:**
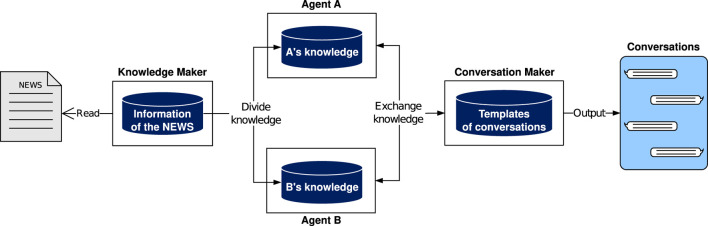
Overview of the entire system. Objective information is extracted from news articles and is distributed to “Agent A” and “Agent B”. Subsequently, “Agent A” and “Agent B” present information from their own knowledge or request information they lack according to the state determined by the “Conversation Maker”. The “Conversation Maker” monitors this and generates conversation sentences by filling in the blanks in the template sentences.

The system diagram is shown in Figure 4. The “Knowledge Maker” extracts eight elements of objective information from existing news articles and distributes the knowledge structure to “Agent A” and “Agent B.” The “Conversation Maker” determines the conversation state based on the state transition model. “Agent A” and “Agent B” follow that state, either presenting information from their own knowledge or requesting information that is not in their knowledge. The “Conversation Maker” monitors this process by the two agents and outputs conversation sentences by filling in the blanks in the template sentences with the facets.

### 3.4 Outputs

Table 1 shows some examples of conversations generated by the proposed system. The system generates conversations in Japanese, so it outputs the sentences in Japanese (the English translations are given in the parentheses). For instance, Example 1 is a conversation that could be outputted when two agents have a distributed knowledge such as that shown on the right-hand side in Figure 2, based on the summarized sentence of an article saying, “Due to the rapidly weakening Yen, the US IT giant, Apple, will raise the price of smartphones in Japan from October 1st.” The state transition goes as “B’s question,” “A’s Answer,” “A’s presentation of information,” “B’s reaction,” etc., The agents express the process of exchanging information as a conversation by presenting the six elements of information they hold or asking about missing information. Example 2 is a conversation that can be outputted when both agents have all the information except “When,” based on the summarized sentence of an article saying, “Facebook Inc. changed the name of their company to ‘Meta’ on 1st.”. The state transition goes as “B’s presentation of information,” “A’s reaction,” “A’s presentation of information,” “B’s reaction,” etc.

**TABLE 1:** Examples of conversation outputted by the proposed system.

Example 1	Example 2
**B:** いつ?	**B:** 変更したらしいね
*(When is it?)*	*(I hear they have made some changes.)*
**A:** 1日からだよ	**A:** らしいね
*(I heard it is from October 1st.)*	*(Looks like it.)*
**A:** 日本でらしいね	**A:** フェイスブックがね
*(It’s in Japan.)*	*(About FaceBook Inc.)*
**B:** うんうん	**B:** そうなんだよね
*(Yes.)*	*(I know.)*
**B:** アメリカのIT大手・アップルがだよね	**A:** 社名をね
*(It’s the US IT giant, Apple, right?)*	*(It is about the name of the company.)*
**A:** そうだよ	**B:** うん
*(Yes.)*	*(Yes)*
**A:** スマホをね	**B:** 「メタ」にだって
*(It is about smartphones.)*	*(To “Meta”,right?)*
**B:** そうだね	**A:** うんうん
*(That’s right.)*	*(Yes,It’s Right.)*
**A:** 値上げしたんだってね	**A:** あれ、いいよね
*(I heard they raised the price.)*	*(That’s cool.)*
**B:** なるほどね	**B:** うん、いい感じだよね
*(I see.)*	*(Yeah, it’s nice.)*
**A:** なんでだろうね	**B:** いつだっけ?
*(I wonder why.)*	*(When is it?)*
**B:** 急激に進む円安の影響でじゃないかな	**A:** 1日だって
*(I think it is due to the rapidly weakening Yen.)*	*(I heard it is from the 1st.)*
**B:** 嫌だな∼	
*(I hate it.)*	
**A:** ちょっと嫌だね	
*(I don’t really like it.)*	

Furthermore, the subjective information that appears in conversations is generated by calculating the average of the preference scale for words included in the news, as mentioned above, and conversations along this average value are outputted. For instance, in Example 1, the preference scale for A is −0.1 and for B is −0.3 toward the news. The system selects sentences from the template sentences in the database and outputs them according to this the preference scale.

## 4 Experiments

The purpose of this research is to investigate the conditions necessary for the medium to continuously convey information, having realized an information conveyance medium with subjective information by two autonomous agents.

As mentioned in Section 1, a lack of consistency in subjective information can cause discomfort and users can reject the medium. Therefore, to realize the medium for information conveyance that expresses information, including subjective information as a conversation, it is necessary that the subjective information of the agent is found to be consistent. As mentioned in Section 3.1.2, in this research, the subjective information of the agent is represented as a preference for words. Therefore, experiments were performed in this work to compare the preferences that the agent has for several words with the preferences that humans have, to confirm whether the subjective information of the agent is as consistent as is the case of humans.

As mentioned in Section 1, in order to convey information continuously, it is necessary to improve the clarity of the information provided by the medium and the sustained attractiveness of the medium.

Regarding the clarity of the information, this aspect must be improved to fulfill the fundamental purpose of an information conveyance medium—accurately conveying existing information. Moreover, if the information is too complex, the recipient may lose interest in the medium and disengage. Therefore, enhancing the clarity of information is crucial for ensuring continuous information conveyance.

Regarding the sustained attractiveness of the medium, this refers to the ability to keep recipients actively engaged and motivated to continue acquiring information. If this aspect is lacking, recipients may abandon the medium before the intended information is fully conveyed.

Thus, from the perspective of continuous information conveyance, this study aims to identify the conditions necessary to ensure that recipients remain motivated to continue receiving information.

**FIGURE 5:**
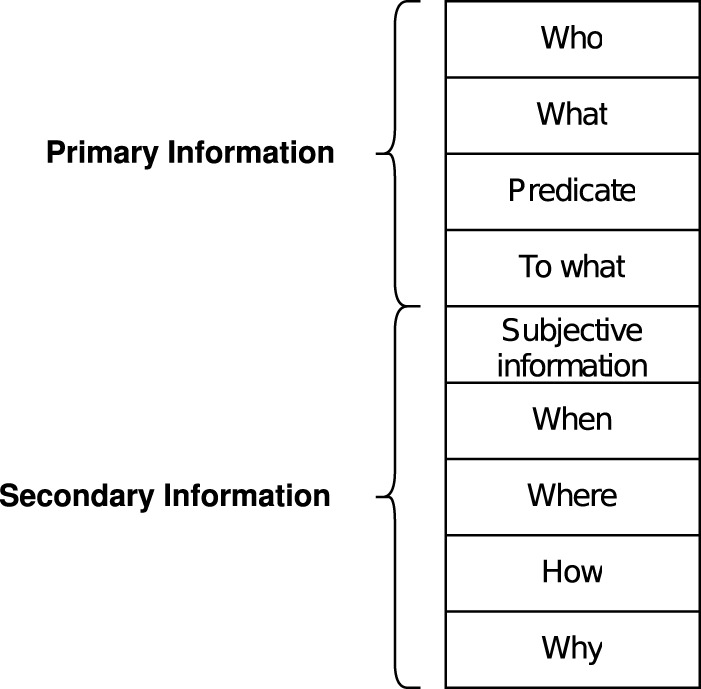
The division of the nine elements of information, which include objective and subjective information, into “Primary Information” and “Secondary Information”. In a sentence, grammatically important elements such as the subject, predicate, object, and complement are classified as “Primary Information”. Through experiments, an investigation has been done on whether the order in which these two types of information are conveyed has any effect on the clarity of the information and the sustained attractiveness of the medium.

To improve the clarity of the information provided by the medium, this research focuses on the order in which information is conveyed. In particular, as shown in Figure 5, the nine elements of information that combine objective and subjective information have been divided into “Primary Information” and “Secondary Information”, and the effect of the order in which the two types of information are conveyed on the clarity of the information have been investigated by asking the subjects to respond to the questions. This method of dividing information is based on grammatically important things in sentences, such as the subject, predicate, object, and complement, as “Primary Information” and anything else as “Secondary Information”.

To improve the sustained attractiveness of the medium, this research focuses on the order of appearance of information in the conversation and the type of media used for conveyance. If unimportant information is conveyed first, the medium might lose interest before the essence of the information is conveyed, which could prevent the information conveyance. With regards to the type of media used for conveyance, it seems that the same conversation can give a different impression depending on the medium used. This research aims to clarify the differences between a text chat conversation by two agents and a conversation by two robots. Robots are more likely to be perceived as having emotions and intelligence (Matsui and Yamada, 2018), which may encourage observers to remain actively engaged. Therefore, we hypothesize that robots have the potential to enhance the sustained attractiveness of the medium compared to text-based chat.Through experiments with subjects, an investigation has been done on the effect of the aforementioned order of information conveyance and the type of media on the sustained attractiveness of the medium.

The experiment to confirm whether the subjective information of the agent is consistent has been detailed in Section 4.1. In addition, the experiments investigating the conditions for conveying information continuously have been detailed in Section 4.2 and Section 4.3.

### 4.1 Experiment for confirming the consistency of the subjective information of the agent

#### 4.1.1 Purpose and hypothesis of the experiment

In the proposed system, the subjective information of the agent is represented by calculating the preference for news based on the preference for words. As detailed in Section 3.1.2, for the agent to get preferences for words, humans manually set preferences for some words in the conceptual network, and these preferences are propagated based on the relationships defined in the conceptual network. In this way, the preferences for other words in the conceptual network are also determined. Therefore, the purpose of this experiment is to validate the following hypothesis:

Hypothesis 1 When the preferences set by humans are automatically propagated, the propagated preferences are similar to those preferences held by the human who set them, and the subjective information of the agent is consistent with that of humans.

#### 4.1.2 Experimental procedure

In order to verify the above-mentioned hypothesis, experiments were conducted following the procedure below, with cooperation from three subjects (both male and female) in their twenties. Initially, each subject assigned a numerical preference scale (“Preference Scale”) ranging from −1.0 to 1.0 to each of the 1,200 words.

Subsequently, these 1,200 words with assigned “Preference Scale” values were randomly divided into two groups: one group consisting of 1,000 words and another group of 200 words. Using the 1,000-word group, the previously described method of propagating “Preference Scale” values across the entire ConceptNet was applied to compute the “Preference Scale” for every word within the network. As a result, it is anticipated that the remaining 200 words not included in this propagation would automatically receive assigned preference scales.

This experiment aimed to examine differences between these automatically computed scales for the 200 words (we call it the “auto-calculated scale”) and the human-assigned preference scales for the same 200 words (we call it the “correct scale”). It is important to note that whether the values of the “auto-calculated scale” are higher or lower than those of the “correct scale” is irrelevant. Instead, the crucial factor is the magnitude of deviation from the human-assigned scales. Therefore, the analysis focused specifically on the differences between the “auto-calculated scale” and the “correct scale.”

Demonstrating how small these auto-calculation differences could be was considered essential. As a benchmark for evaluating the magnitude of differences, a comparison was made with differences resulting from randomly assigning preference scales (we call it “random scale”)

#### 4.1.3 Results and discussion

**FIGURE 6:**
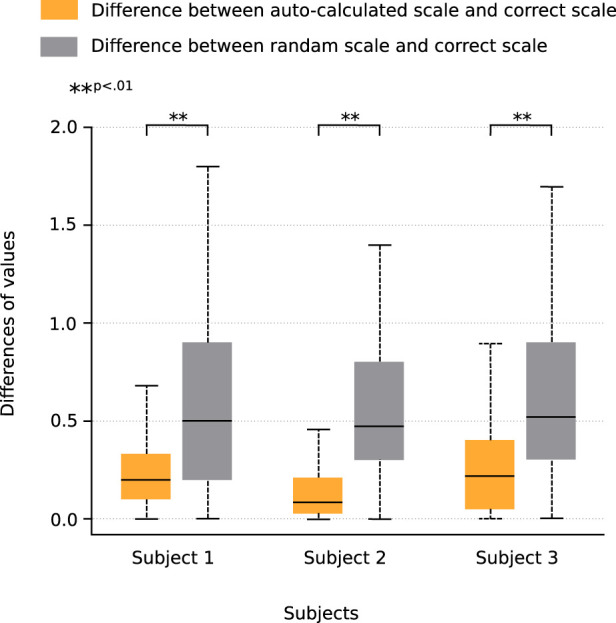
Results of the experiment performed for investigating whether the agent is acquiring consistent subjective information. For words where the preference scale has been auto-calculated, the difference between the “correct scale” and “auto-calculated scale”, and the difference between the “correct scale” and “random scale” are compared.

Figure 6 is shows the difference between the “correct scale” and the “auto-calculated scale,” and the difference between the “correct scale” and the “random scale” for each of the 200 words for the three subjects. However, not all words in the conceptual network may be connected to each other. Thus, some words, which were selected randomly through the procedure in Section 4.1.2, may not be propagated and automatically calculated. The results in Figure 6 exclude words for which the preference scale was not propagated due to the structure of the conceptual network, and thus the difference could not be calculated. The orange graph represents the difference between the “correct scale” and the “auto-calculated scale,” whereas the gray graph represents the difference between the “correct scale” and the “random scale.” In addition, for each subject, the average values were compared between the two groups statistically using the Wilcoxon signed-rank sum test. As a result, a significant difference (p<.01) was observed in the average values between the two groups for all three subjects.

These results show that the difference between the “auto-calculated scale” and the “correct scale” is significantly smaller than the difference between the “random scale” and the “correct scale,” which suggests that the preference scale values that is automatically calculated through propagation are closer to the correct values assigned by the subjects compared to random values. In addition, since only 1.05% of the differences between the “auto-calculated scale” and the “correct scale” exceeds 1.0, it is considered that the preferences determined by automatic calculation through propagation demonstrate preferences similar to humans and possess the same level of consistency.

### 4.2 Experiment for investigating the effects of the order of information on the clarity of the information

#### 4.2.1 Purpose and hypothesis of the experiment

For the medium to convey information continuously, it needs to achieve the original purpose of an information conveyance media to convey the information properly. Furthermore, by conveying essential information first, the most important parts of the news make an impression on the users and the overall information becomes easier to understand. Therefore, the order in which information is conveyed can affect the clarity of the information. Hence, in this experiment, the information was divided into two groups: “Primary Information” and “Secondary Information.” The purpose of this experiment was to investigate whether the order of conveying these two types of information affect the clarity of the information, and verify the following hypothesis:

Hypothesis 2 Conveying the “Primary Information” first makes the information easier to understand in the case of text-based conversation.

#### 4.2.2 Experimental procedure

**FIGURE 7:**
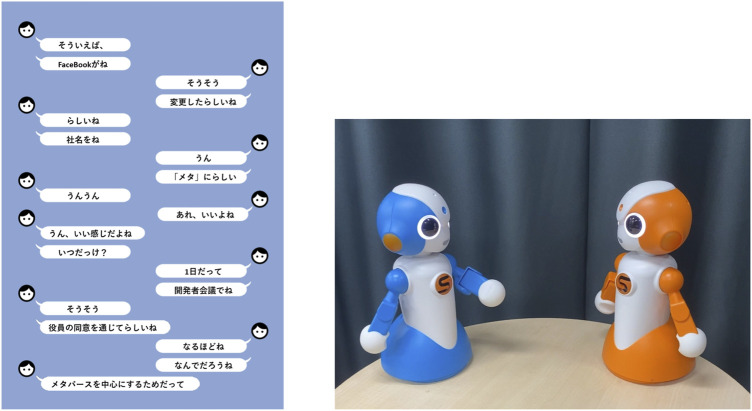
Conversation outputs used in the experiment in a message chat format (left) and a robot conversation format (right). The subjects watched videos of both formats.

In order to verify Hypothesis 2, an experiment was performed with 100 native Japanese speakers, involving 57 males and 43 females ranging between 21 and 65 years old. After watching videos of conversations between two agents in a message chat format, as shown on the left-hand side of Figure 7, the subjects answered a questionnaire. The conversation where the “Primary Information” was presented first and the conversation where the “Secondary Information” was presented first are shown in Table 2. Since the experiment was conducted entirely in Japanese, we provide actual conversation examples along with their English translations. In this experiment, the order of conveying information within the “Primary Information” or “Secondary Information” was kept fixed. In particular, “Who,” “Predicate,” “What,” and “To what” were presented in order within the “Primary Information”, whereas the “Subjective information”, “When,” “Where,” “How,” and “Why” were presented in order within the “Secondary Information,” as described in Figure 5.

**TABLE 2:** The conversation used in the experiment. The subjects watched these conversations in message chat format or as two robots chatting.

Primary information first	Secondary information first
**A:** そういえば	**A:** そういえば
*(By the way,)*	*(By the way,)*
**A:** フェイスブックがね	**B:** あれ、いいよね
*(About Facebook, Inc.)*	*(That’s cool.)*
**B:** そうそう	**A:** うん、いい感じだよね
*(Oh yes.)*	*(Yeah, it’s nice.)*
**B:** 変更したらしいね	**A:** いつだっけ?
*(I hear they have made some changes.)*	*(Since when?)*
**A:** らしいね	**B:** 1日だって
*(I heard too.)*	*(I heard it is from 1st.)*
**A:** 社名をね	**B:** 開発者会議でね
*(It is about the name of the company.)*	*(At the developers’ conference, isn’t it?)*
**B:** うん	**A:** そうそう
*(Yes)*	*(Yes)*
**B:** 「メタ」にらしい	**A:** 役員の同意を通じてらしいね
*(To “Meta”,right?)*	*(Through executive consent, I hear.)*
**A:** うんうん	**B:** なるほどね
*(Yes, that’s right.)*	*(I see.)*
**B:** あれ、いいよね	**B:** なんでだろうね
*(That’s cool.)*	*(I wonder why.)*
**A:** うん、いい感じだよね	**A:** メタバースを中心にするためだって
*(Yeah, it’s nice.)*	*(It’s to make the Metaverse the center of their business.)*
**A:** いつだっけ?	**A:** フェイスブックがね
*(Since when?)*	*(About Facebook, Inc.)*
**B:** 1日だって	**B:** そうそう
*(I heard it is from the 1st.)*	*(Oh yes.)*
**B:** 開発者会議でね	**B:** 変更したらしいね
*(At the developers’ conference, isn’t it?)*	*(I hear they have made some changes.)*
**A:** そうそう	**A:** らしいね
*(Yes)*	*(I heard too.)*
**A:** 役員の同意を通じてらしいね	**A:** 社名をね
*(Through executive consent, I hear.)*	*(It’s about the name of the company.)*
**B:** なるほどね	**B:** うん
*(I see.)*	*(Yes)*
**B:** なんでだろうね	**B:** 「メタ」にらしい
*(I wonder why.)*	*(To “Meta”,right?)*
**A:** メタバースを中心にするためだって	**A:** うんうん
*(It’s to make the Metaverse the center of their business.)*	*(Yes,that’s right.)*

To evaluate the clarity of the information, the question “Was the information easy to understand?” was asked after the subjects had watched the conversation. The subjects answered this question using the 7-point Likert scale.

#### 4.2.3 Results and discussion

**FIGURE 8:**
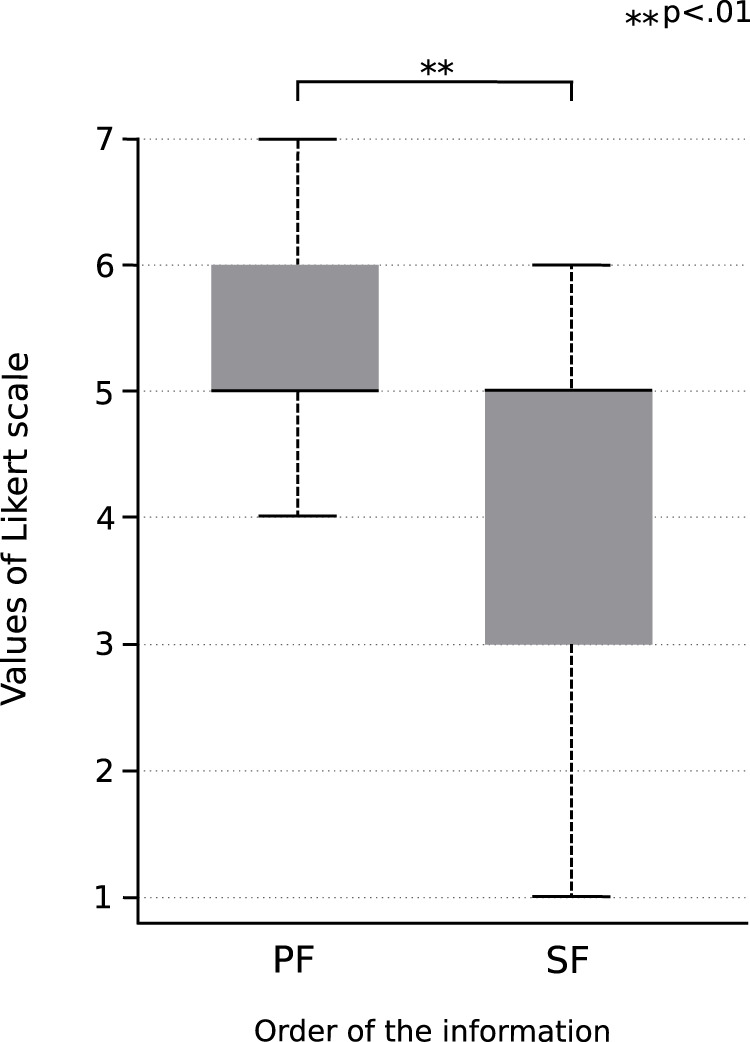
Results of the experiment performed for investigating the effect of the order of presenting information on the clarity of the information. “PF” is the result of the conversation in which the “Primary Information” was presented first, whereas “SF” is the result of the conversation in which the “Secondary Information” was presented first.

Figure 8 shows the comparison of the clarity of the information between the conversation where the “Primary Information” was presented first and the conversation where the “Secondary Information” was presented first. The former has been labeled as “PF” and the latter as “SF”. When comparing the averages between the two groups (PF and SF) using the Wilcoxon signed-rank sum test, a significant difference (p<.01) was observed between the average values of the two groups.

Therefore, in the case of conversations in the message chat format, it is clear that presenting the “Primary Information” before the “Secondary Information” improves the clarity of the information compared to when “Secondary Information” is presented first.

### 4.3 Experiment for investigating the effect of the order of information and the type of media on the sustained attractiveness of the medium

In the proposed medium, where the receivers of information do not interact, i.e. they are passive, it is necessary to make them interested in the medium in order for them to receive the information. Furthermore, if the medium cannot maintain their interest, they may quickly lose interest and the medium may be unable to convey the information. Therefore, it is important for the medium to constantly attract people in order to convey information continuously.

To improve this sustained attractiveness of medium, this study focused on the order of information conveyed and the type of media. It is considered that conveying unimportant information, such as the “Secondary Information”, first could make it harder to understand what kind of information is being conveyed. This can make the receiver lose interest easily and affect the sustained attractiveness of the medium. Furthermore, we also focused on the type of media because it is considered that media that can convey information multimodally like robots can represent intelligence and emotions more easily, thus making them more attractive.

Therefore, in this experiment, the conditions for improving the sustained attractiveness of the medium were verified by validating the following hypotheses:

Hypothesis 3 Presenting the “Primary Information” first improves the sustained attractiveness.

Hypothesis 4 Conversations using robots improves the sustained attractiveness more than conversations using text.

#### 4.3.1 Experimental procedure

Similar to the experiment in 4.2, we conducted with 100 native Japanese speakers involving 57 males and 43 females ranging between 21 and 65 years. The subjects watched two conversations, namely, the “Primary Information” first conversation and “Secondary Information” first conversation shown in Table 2, in a message chat format, shown on the left-hand side of Figure 7 and the conversation with the robot, shown on the right-hand side of Figure 7. They watched these four patterns of conversation in video format and answered a questionnaire.As with the other experiments, the experiment was conducted entirely in Japanese.

#### 4.3.2 Questionnaire

In order to measure the sustained attractiveness of the medium, three questions were prepared for the subjects to answer. Below is a detailed explanation about why these three questions were necessary for measuring the sustained attractiveness of the medium.

• Q1. How much did you like the conversation of the two agents?

Since the system is conveying information through the conversation of two agents, the agents are the media themselves. This question was asked since the subjects liking the two agents equate to feeling of the medium being attractive.

•Q2. How boring was the conversation of the two agents?

If the conversation between the two agents is boring, the subjects are more likely to lose interest in this medium. To enhance sustained attractiveness, it is necessary not to bore the subjects. This question was asked to measure whether this medium bore the subjects.

•Q3. How interested were you in their conversation?

When a conversation attracts interest, it is likely for the subjects to want to listen to more of it. Therefore, in order to convey information continuously, it is necessary to attract interest through conversation. Thus, this question was asked.

The subjects answered the above three questions on a 7-point Likert scale for each of the four conversation patterns. However, for the question of boredom, the scoring range was set such that 1 is corresponds to most boring and 7 corresponds to is not boring at all.

#### 4.3.3 Results and discussion

**FIGURE 9:**
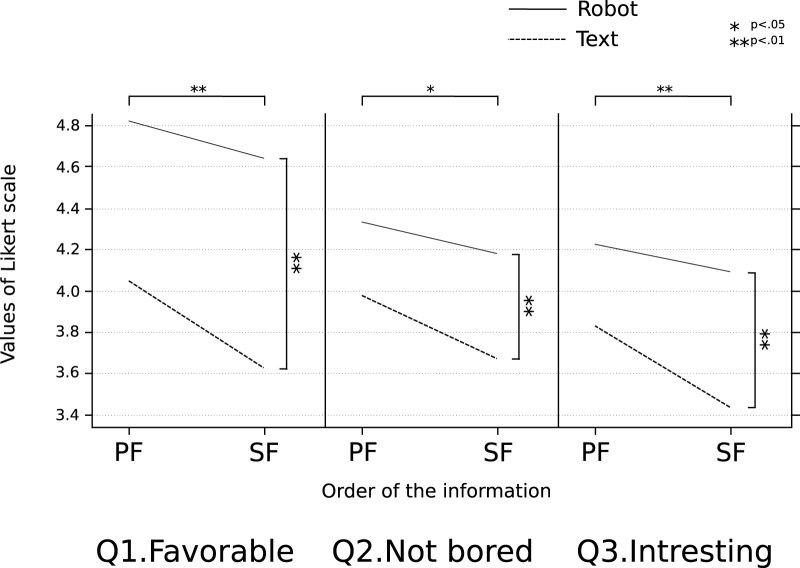
Results of the experiment performed for investigating the effect of the order of conveyed information and the type of media on the sustained attractiveness. The interaction diagram shows three indicators when subjects answered the questionnaire on the Likert scale. “PF” is the conversation where “Primary Information” was presented first, and “SF” is the conversation where “Secondary Information” was presented first.

Figure 9 shows the results of the experiment in the form of graphs for the three questions that the subjects answered. Each graph represents the 2 × 2 interaction between the robots and text, and the conversation where “Primary Information” was presented first *versus* the conversation where “Secondary Information” was presented first. For the question about boredom, since the value decreases if it is boring, it is denoted as “Not bored” in the figure. The results of the two-factor analysis of variance (ANOVA) within subjects for the three questions are described below in detail. The robot *versus* text has been denoted as the media factor, and the conversation in which “Primary Information” was presented first *versus* the conversation where “Secondary Information” was presented first has been denoted as the order factor.

First, for the question “Q1. How much did you like the conversation of the two agents?,” a significant difference was observed for the media factor (F(1,99)=54.35,p<.001) and the order factor (F(1,99)=11.8,p<.001) , whereas no significant difference was observed for the interaction (F(1,99)=2.006,p=0.16) .

Next, for the question “Q2. How boring was the conversation of the two agents?”, a significant difference was observed for the media factor (F(1,99)=12.24,p<.001) and the order factor (F(1,99)=3.953,p=0.0495) , but no significant difference was observed for the interaction (F(1,99)=0.501,p=0.481) .

Finally, for the question “Q3. How interested were you in their conversation?”, a significant difference was observed for the media factor (F(1,99)=16.4,p<.001) and the order factor (F(1,99)=7.949,p=0.0058) , but no significant difference was observed for the interaction (F(1,99)=2.202,p=0.141) .

Significant differences were observed in the media and order factors for all three questions, whereas no interaction was observed between the two factors. From this, it is clear that the conversations where “Primary Information” is presented first can improve the sustained attractiveness of the medium more than the conversations where “Secondary Information” is presented first. Furthermore, it was clarified that robots can improve the sustained attractiveness of the medium more than text.

Additionally, as detailed in Section 4.3.2, the Question 2 and Question 3 were designed with distinct roles to measure the sustained attractiveness of the medium. However, Spearman’s rank correlation analysis revealed a significant positive correlation between the responses to these two questions $\left(Spearman^{\prime }s\;rho=0.644,p<.001\right)$ .These results suggest that “interest” and “not being bored” are interrelated concepts and that the two question items may share similar characteristics.

### 4.4 Inferences from the experiments

As mentioned in Section 4.1, the experiment investigating whether the subjective information of the agents is consistent verifies that the preferences automatically calculated and determined by propagation are similar to those of humans and have the same level of consistency as humans. Therefore, Hypothesis 1 is verified. In addition, from the results of the experiment investigating the effect of the order of information on the clarity of the information, discussed in Section 4.2, it is clear that the conversation giving “Primary Information” first significantly improves the clarity of the information compared to the conversation giving “Secondary Information” first. Therefore, Hypothesis 2 is verified. Furthermore, from the results of the experiment investigating the effect of the order of the information and the type of media on the sustained attractiveness of the medium, discussed in Section 4.3, it is clear that conversations giving “Primary Information” first and the conversation among robots significantly improves the sustained attractiveness of the medium compared to the conversation giving “Secondary Information” first and the conversation by text. Therefore, Hypothesis 3 and Hypothesis 4 are verified.

Therefore, the two agents are able to achieve the consistency of subjective information necessary for the realization of information conveyance medium involving subjective information. It has also been shown that this medium needs to take at least the order of information conveyance and the type of media into account as conditions necessary for continuous information conveyance.

## 5 Conclusion

In this research, the information conveyance medium involving subjective information has been realized using two autonomous agents. The two agents, which have knowledge constructed from existing news and conceptual networks express the process of exchanging information they hold as a conversation. Toward the realization of this medium, experiments were performed to confirm whether the two agents have consistent and natural subjective information. To investigate the conditions necessary for this medium to convey information continuously, we focused on the clarity of the information provided by the medium and the sustained attractiveness of the medium in this study, and conducted two experiments.

From the results of the experiments, it was shown that the agents can autonomously construct consistent subjective information if humans set part of the subjective information of the agent. It was also shown that one of the conditions for improving the clarity of information provided by the medium is to convey the grammatically important information first, such as the subject, predicate, object, and complement, as shown in Figure 5. Furthermore, two conditions for improving the sustained attractiveness of the medium, namely, to convey important information first and to output conversation via robots, not text, have been verified.

Therefore, a medium where two autonomous agents with consistent opinions convey information while including subjective information through a conversation has been realized in this study. In addition, this type of information conveyance medium using conversation between two agents has revealed the two conditions necessary for continuous information conveyance: the order of information and the type of media.

### 5.1 Limitations

As mentioned in Section 4.1.3, in this research, the conceptual network was used and the preference scale was propagated. However, in some cases, some words might have fewer connections with other words and form a closed network, in which case the preference scale is not propagated. Ideally, once humans have assigned the preference scale to some words, the other words should automatically be assigned. However, in this experiment, 79% of the words were automatically calculated through propagation, whereas the remaining 21% of words had no connections with other words and the preference scale was not propagated. This could be because the conceptual network we created was small and there were many words with fewer connections to other words. Therefore, if the conceptual network is enlarged and increases the connection between words, any given word can always be reached by following a connection from another word via other connecting words. Then, this problem can be resolved.Although the experiment in Section 4.1 involved only three subjects, approximately 200 data points were collected from each participant. Given the substantial total data collected, statistical analyses are considered sufficiently robust. However, conducting experiments with a larger number of subjects would further improve the generalizability of the findings.In this study, we defined the “Primary Information” and “Secondary Information” and investigated the difference in impressions based on their order of conveyance in the conversation. However, since the order of conveyance within “Primary Information” and “Secondary Information” was fixed in all conversations, the effect of these differences in order were not verified. In addition, we only performed an investigation on one topic, as shown in Figure 2, and thus the effect of different topics were not verified. Investigations on other factors, such as the difference in the order of conveyance of detailed information and the difference according to the topic, can show a general contribution in future studies.Furthermore, the experiments in this study were conducted in Japanese, and all conversations and questionnaire items used in the experiments described in this paper were translated from the original Japanese into English. While we took great care to ensure the accuracy of the translations, it is not always possible to perfectly reproduce Japanese-specific concepts and expressions in English. This limitation may influence how readers interpret the study, and therefore, it should be taken into account when evaluating the results.For future research, it will be necessary to validate the accuracy of translations and consider conducting experiments in multiple languages to ensure greater consistency across languages.Additionally, in this study, experiments were conducted using videos of robot conversations rather than employing physically embodied robots. Consequently, the robots’ physical presence could not be accounted for, making it difficult to clearly evaluate the effects of embodiment on information conveyance and human cognition or behavior based on this study’s results. Therefore, when applying the findings of this research to physically embodied robots, the potential effects of embodiment on the outcomes must be considered. Furthermore, embodiment differences may influence direct comparisons with previous studies. Future research should thus implement the same tasks using both physically embodied robots and robot videos to comparatively investigate these differences, thereby clarifying in greater detail how embodiment impacts information conveyance.Lastly, in this research, we extracted eight elements of information after summarizing news articles. Thus, there is a possibility of failing to extract the information depending on the summarized sentences. In particular, if multiple sentences are connected to construct one sentence, or if a sentence is output with multiple “Predicate” information, it can be difficult to extract the information even if the summarized sentence is sufficiently natural. Currently, we only convey information when we can successfully obtain it, but to solve this problem, some improvements are required, such as using large language models in extracting the eight elements so that we can directly extract the eight elements of information from the original news article.

### 5.2 Future work

The medium realized in this research has been investigated for conditions for continuous information conveyance in experiments within the laboratory. However, the actual use of such media is in the real world. Therefore, in the future, we aim to perform a field investigation on how much the psychology and behavior of observers can be changed when two agents convey the information.

In addition, although subjective information has been represented by a single value from −1.0 to 1.0 in this research, the subjective information of humans is complex and cannot be expressed on a single axis. By using emotion models, such as Russell’s Model (Russell, 1980), more flexible conversations, which do not use only template sentences, can be achieved by expanding the subjective information axis. Therefore, we aim to realize two agents having this type of complex subjective information.

## Data Availability

The raw data supporting the conclusions of this article will be made available by the authors, without undue reservation.
